# Supplementary material for: A machine learning approach for computation of cardiovascular intrinsic frequencies
Source: PLoS One. 2023 Oct 26;18(10):e0285228. doi: 10.1371/journal.pone.0285228 (PMC10602266; doi:10.1371/journal.pone.0285228)
Supplement: Attachment — Submitted filename: Response to Rewievers.docx [file pone.0285228.s001.docx]

We sincerely thank the editor and reviewers for their comments and suggestions. Below we list our point-by-point responses in the sequence in which they were raised in each reviewer’s report. The corresponding changes and additions to the manuscript are marked and provided in italic *blue*.

**Journal Requirements:**

When submitting your revision, we need you to address these additional requirements.

1. Please ensure that your manuscript meets PLOS ONE's style requirements, including those for file naming. The PLOS ONE style templates can be found at

https://journals.plos.org/plosone/s/file?id=wjVg/PLOSOne_formatting_sample_main_body.pdf and https://journals.plos.org/plosone/s/file?id=ba62/PLOSOne_formatting_sample_title_authors_affiliations.pdf

**Response**: We have ensured that the manuscript conforms to PLOS ONE’s style requirements.

2. Thank you for stating the following in the Competing Interests section:

"Niema M. Pahlevan holds equity in Avicena LLC and has a consulting agreement with Avicena LLC. The remaining authors do not declare any competing interest."

Please confirm that this does not alter your adherence to all PLOS ONE policies on sharing data and materials, by including the following statement: "This does not alter our adherence to PLOS ONE policies on sharing data and materials.” (as detailed online in our guide for authors http://journals.plos.org/plosone/s/competing-interests). If there are restrictions on sharing of data and/or materials, please state these. Please note that we cannot proceed with consideration of your article until this information has been declared.

Please include your updated Competing Interests statement in your cover letter; we will change the online submission form on your behalf.

**Response**: "This does not alter our adherence to PLOS ONE policies on sharing data and materials.”. The competing interest statement is included in the cover letter.

**Reviewers’ Comments:**

**Reviewer #1:**

1. Please show the computational time required for the optimization method as compared to the machine learning approach.

**Response:**

The computational time required for the optimization method was added and compared with the machine learning’s time in the manuscript.

**(Section 4, Paragraph 4)**: “*The online predictions are almost real-time for each resampled waveform in blind clinical tests (3.94e-5 seconds on a laptop with a 2.3 GHz Quad-Core Intel Core i5 CPU). The offline training takes around 14 minutes per network (100% training dataset; maximum 5 layers; 512, 256, 128, 64 and 32 possible neurons; one restart*). *In contrast, the L_2_ optimization for IF computations takes multiple seconds per waveform (around 9 to 40 seconds depending on the optimization settings via the same laptop).”*

2. A lot of relevant and interesting figures are shown in the results section. It would be good if the author highlight the key information from the figures.

**Response:**

More details and information about the figures were added to the text of the manuscript in the “Results” section:

**(Section 3.2, Paragraph 1)**:

“*The 100% of the training data corresponds to N=13,008 waveforms. It is noteworthy that MSE is in absolute units corresponding to its calculation from the normalized outputs.”*

**(Section 3.3, Paragraph 1)**:

“*Here, we use the blind clinical test to assess the efficacy of our proposed ML-based IF model with different indices and figures. The resulting RMSE and relative errors of the blind clinical test (described in Section 2.7) are presented in Table 3. To investigate and confirm the agreement between the FNN-based IF parameter predictions and the standard L_2_-based IF calculations, we also used blind test data and plotted different statistical analysis figures (i.e., regression plots, Bland-Altman plots, and error histograms) for the scaled IF parameters (Fig. 5 and Fig. 6). The diagonal dotted line in the regression plots represents equality. In the Bland-Altman plots, limits of agreement (LoA) are shown by the two horizontal solid lines in purple. The Bland-Altman analysis helps with confirming a low bias for the ML-based IF parameter predictions. The error histograms also help with investigating and confirming low occurrence of high errors for the ML-based IF parameter predictions.”*

**(Section 3.4, Paragraph 1)**:

“*As mentioned earlier, the ML model requires that every waveform undergoes a normalization and a resampling to 500 samples (datapoints) per cardiac cycle. However, the model dependency on the original sampling rate of the waveform or the measurement device should be assessed to ensure that the model stays accurate across different sampling rates and measurement devices.”*

**(Section 3.4, Paragraph 1)**:

“*The vector size of these waveforms ranges from 50 to 169 (for iPhone measurements), 150 to 619 (for Tonometry measurements), and 558 to 1560 (for Vivio measurements). The box-whisker plot here displays a five-index summary of error for each device by showing the minimum, first quartile, median, third quartile, and maximum.”*

**(Section 3.4, Figure 7 & 8 captions)**:

“*The measurement devices include Tonometry (shown by blue circles), Vivio (shown by red circles), and iPhone (shown by orange circles).”*

E. Discussion:

1. Although the Machine learning approach in principle shows comparable performance to the optimization approach, it would be interesting if the author can explain in what condition the system will perform badly as some of the data shows very poor performance.

**Response**: After looking at Figure 7 and Figure 8, and also looking at the distribution of the errors, it is noticed that most of the relatively high errors are related to the waveforms with lower original vector sizes. So, although the model performed very well for majority of low vector size waveforms, one reason for high error could be due to over-sampling of the waveforms during preprocessing. The following sentence were added to the manuscript:

**(Section 4, Paragraph 3)**:

“*Although the model performed very well for majority of waveforms with low original vector size, over-sampling of the waveforms during preprocessing can cause error as most of the relatively high errors occurred for the waveforms with lower original vector sizes.”*

_______________________________________________________________

**Reviewer #2**:

1. Section 1 and Section 2: Description of Intrinsic Frequency (IF) method described in Section 1 and Section 2 has some repetitions and can be combined and focus in one of the sections only. Introduction can give a brief description of this method.

**Response:**

Sections 1 and 2 have been modified according to this comment. The Introduction section has been modified to address this comment.

**(Section 2.1, Paragraph 2)**: “*When applied to arterial waveforms, the IF method reveals clinically important information about the dynamics of the LV and the arterial system (as well as their interactions) in both healthy and disease conditions [21, 23, 24, 28-33]. Several clinical studies have confirmed the clinical usefulness of cardiovascular IFs in both the diagnosis and prognosis of cardiovascular diseases (CVDs) [23, 28, 30, 34]. One important advantage of the IF method for clinical applications is that the absolute magnitude of the arterial pressure wave is not required to extract the IF parameters; only the waveform morphology is needed [20, 28]. As such, IFs can be easily acquired noninvasively and inexpensively using a smartphone [24, 28], arterial applanation tonometry, or a wearable sensor. In a notable study, the left ventricle ejection fraction (LVEF) was accurately approximated by applying the IF method to the noninvasive carotid waveforms measured by an iPhone camera [28].”*

**(Section 2.1.2, Paragraph 2)**: *“The systolic IF* parameters (*ω_1_,* $\varphi_{1}, R_{s}$*) are dominated by the dynamics of the coupled LV-arterial system, whereas the diastolic IF parameters (ω_2_,* $\varphi_{2}, R_{d}$*) are dominated by the dynamics of just the arterial network [23, 24, 28, 30, 32, 38-41]. Figure 1B illustrates details about the above-mentioned mathematical reformulation through a visualization of* $\omega_{1}$*,* $\omega_{2}$*,* $\varphi_{1}$*,*$\varphi_{2}$*,* $R_{s}$ *and* $R_{d}$ *during the systolic and diastolic phases.”*

2. Section 3 Results: Brief descriptions were provided for the results. The authors should elaborate more about the results presented in each table and figure. Beside using Figure, it will be good to have a table to show the comparison of results computed by the FNN model and L2 Optimization-based method.

**Response:**

a) More details and information about the results were added to the manuscript in the “Results” section:

**(Section 3.2, Paragraph 1)**:

“*The 100% of the training data corresponds to N=13,008 waveforms. It is noteworthy that MSE is in absolute units corresponding to its calculation from the normalized outputs.“*

**(Section 3.3, Paragraph 1)**:

“*Here, we use the blind clinical test to assess the efficacy of our proposed ML-based IF model with different indices and figures. The resulting RMSE and relative errors of the blind clinical test (described in Section 2.7) are presented in Table 3. To investigate and confirm the agreement between the FNN-based IF parameter predictions and the standard L_2_-based IF calculations, we also used blind test data and plotted different statistical analysis figures (i.e., regression plots, Bland-Altman plots, and error histograms) for the scaled IF parameters (Fig. 5 and Fig. 6). The diagonal dotted line in the regression plots represents equality. In the Bland-Altman plots, limits of agreement (LoA) are shown by the two horizontal solid lines in purple. The Bland-Altman analysis helps with confirming a low bias for the ML-based IF parameter predictions. The error histograms also help with investigating and confirming low occurrence of high errors for the ML-based IF parameter predictions.”*

**(Section 3.4, Paragraph 1)**:

“*As mentioned earlier, the ML model requires that every waveform undergoes a normalization and a resampling to 500 samples (datapoints) per cardiac cycle. However, the model dependency on the original sampling rate of the waveform or the measurement device should be assessed to ensure that the model stays accurate across different sampling rates and measurement devices.”*

**(Section 3.4, Paragraph 1)**:

“*The vector size of these waveforms ranges from 50 to 169 (for iPhone measurements), 150 to 619 (for Tonometry measurements), and 558 to 1560 (for Vivio measurements). The box-whisker plot here displays a five-index summary of error for each device by showing the minimum, first quartile, median, third quartile, and maximum.”*

**(Section 3.4, Figure 7 & 8 captions)**:

“*The measurement devices include Tonometry (shown by blue circles), Vivio (shown by red circles), and iPhone (shown by orange circles).”*

b) Also, Table 2 and Table 3 present the comparison of the results computed by the FNN model and the L_2_ optimization-based method:

***Table 3.*** *Errors and ranges of the blind clinical test results for proposed ANN design (N=3009).*

| ***Output*** | ***Range*** | ***RMSE***  *(ML-based Vs. Optimization-based Approach)* | ***Relative Error of the Ensemble***  *(ML-based Vs. Optimization-based Approach)* |
| --- | --- | --- | --- |
| $\hat{\omega}_{1}$ | *[75.0, 155.2]* | *1.81* | *1.82%* |
| $\hat{\omega}_{2}$ | *[19.2, 71.3]* | *2.70* | *5.62%* |
| $\hat{R}_{s}$ | *[0.43, 0.89]* | *0.0139* | *2.01%* |
| $\hat{\varphi_{1}}$ | *[-1.269, -0.008]* | *0.0349* | *9.99%* |
| $\hat{c}$ | *[0.18, 0.58]* | *0.0144* | *4.00%* |

***Table 2.*** *Generalization accuracy results for the optimal ANN model.*

| ***Output*** | ***Range*** | ***RMSE***  *(ML-based Vs. Optimization-based Approach)* | ***Relative Error of the Ensemble***  *(ML-based Vs. Optimization-based Approach)* |
| --- | --- | --- | --- |
| $\hat{\omega}_{1}$ | *[60.4, 143.5]* | *0.63* | *0.71%* |
| $\hat{\omega}_{2}$ | *[26.9, 152.3]* | *1.49* | *2.44%* |
| $\hat{R}_{s}$ | *[0.49, 0.94]* | *0.006* | *0.84%* |
| $\hat{\varphi_{1}}$ | *[-1.06, 0.16]* | *0.014* | *4.74%* |
| $\hat{c}$ | *[0.14, 0.51]* | *0.005* | *1.55%* |

3. Section 3 Results and Section 4 Discussion: Besides correlate your model to the L2 optimization methods, the authors should also explain the performance of the proposed model based on the evaluation metrics. For example, RMSE for some IF parameters is more than 1. Generally, the lower the value of the RMSE, the better the model is. RMSE more than 1 means that a model couldn't find a solution or (optimized very well).

**Response:** The RMSE values (presented in Table 2 and Table 3) are corresponding to the final outputs of the model. RMSE is dependent on the absolute values and ranges of the parameters. Our final output parameters are from different ranges and values, and some have “order of magnitude” difference, so they should be interpreted according to their range. For example, as shown in Table 2, $\hat{\omega}_{1}$ computed from the designed FNN-based IF model results in an RMSE of 0.6 (over a range of 60 to 143) with a relative error of 0.7%. For $\hat{\omega}_{2}$, RMSE was 1.49 (over a range of 27 to 152) with the relative error of 2%. Here, RMSE=1.49 is an acceptable value over a wide range of [27, 152], but of course the same RMSE can never be acceptable for c ̂ with a small range of [0.14,0.51]. The below sentences are related to the discussion of the model performance based on the evaluation metrics in the manuscript:

**(Section 4, Paragraph 2)**: “*As shown in Table 2,* $\hat{\omega}_{1}$ *computed from the designed FNN-based IF model results in an RMSE of 0.6 (over a range of 60 to 143) with a relative error of 0.71%. The corresponding relative error for*  $\hat{\omega}_{2}$ *was 2.4% with an RMSE=1.49 (over a range of 27 to 152).”*

**(Section 4, Paragraph 2)**: *“We have also performed an external (stratified) blind test on the model using a random assortment of clinical pressure waveforms from FHS clinical database that were set aside before constructing and training the model. The resulting correlation coefficients in this blind test were R=0.98 for the first IF (*$\hat{\omega}_{1}$*) and R=0.93 for the second IF (*$\hat{\omega}_{2}$*). The correlation coefficients are higher than R=0.95 for all other IF-related parameters (other outputs). Such high correlations attest to the efficacy of our proposed ML-based IF model. As shown in Table 3, the intrinsic frequencies*  $\hat{\omega}_{1}$ *and*  $\hat{\omega}_{2}$ *demonstrate low relative errors of 1.82% (RMSE=1.81; range: 75-155) and 5.62% (RMSE=2.70; range: 19-71), respectively. The Bland-Altman plots of Fig. 5 and Fig. 6 additionally confirm the excellent agreement between the FNN-based IF parameter predictions and the standard L2-based IF calculations.”*

4. Section 4 Discussions / Section 5 Conclusions: The main limitations found during the test/experiment should be discussed and any possible solution that could be applied to address these limitations.

**Response:**

A new section (Section 4.1) has been added to the manuscript as “Limitations and Future Work”. The following paragraph was included in that section:

**(Section 4.1, Paragraph 1)**: “*The main limitation for the current study is that the clinical value of the IF parameters predicted by our proposed FNN-based approach (for diagnostic and prognostic cardiovascular diseases) has not been assessed. This will be studied in our future work. Furthermore. the methodology proposed in this work will be extended and applied to other complex systems with multiple (two or more) decoupling times.”*

5. Figure 7 and Figure 8 - the table legend used to indicate the color for each device is not very clear, it is hard to know what color is used in the diagram to represent each device. The authors may need to use word to explain what color is used to represent a device.

**Response:**

The captions of figure 7 and figure 8 were modified, and the color information corresponding to each measurement device is added to the manuscript text.

**(Section 3.4, Figure 7)**:

***“Figure 7: Effects of the original sampling rate and measurement device on predictions of IFs from the ANN model.*** *Scatter plots and box-whisker plots of the error for the scaled IFs* $\hat{\omega}_{1}$ *(top row:* ***a1*** *and* ***a2****, respectively) and* $\hat{\omega}_{2}$ *(bottom row:* ***b1*** *and* ***b2****, respectively). The measurement devices include Tonometry (shown by blue circles), Vivio (shown by red circles), and iPhone (shown by orange circles).”*

**(Section 3.4, Figure 8)**:

***“Figure 8: Effects of the original sampling rate and measurement device on predictions of related IF parameters from the ANN model.*** *Scatter plots and box-whisker plots for the scaled first intrinsic envelope (*$\hat{R}_{s}$*; top row:* ***a1*** *and* ***a2****, respectively), the scaled first intrinsic phase (*$\hat{\varphi_{1}}$*; middle row:* ***b1*** *and* ***b2****, respectively), and the scaled fitting constant (*$\hat{c}$*; bottom row:* ***c1*** *and* ***c2****, respectively). The measurement devices include Tonometry (shown by blue circles), Vivio (shown by red circles), and iPhone (shown by orange circles).”*
